# The complement system in age-related macular degeneration

**DOI:** 10.1007/s00018-021-03796-9

**Published:** 2021-03-09

**Authors:** Angela Armento, Marius Ueffing, Simon J. Clark

**Affiliations:** 1 Department for Ophthalmology, Institute for Ophthalmic Research, Eberhard Karls University of Tübingen, Tübingen, Germany; 2 Department for Ophthalmology, University Eye Clinic, Eberhard Karls University of Tübingen, Tübingen, Germany; 3 Lydia Becker Institute of Immunology and Inflammation, Faculty of Biology, Medicine and Health, University of Manchester, Manchester, UK

**Keywords:** Age-related macular degeneration, Ophthalmology, Complement system, Genetics, Ageing, Retinal biology

## Abstract

Age-related macular degeneration (AMD) is a chronic and progressive degenerative disease of the retina, which culminates in blindness and affects mainly the elderly population. AMD pathogenesis and pathophysiology are incredibly complex due to the structural and cellular complexity of the retina, and the variety of risk factors and molecular mechanisms that contribute to disease onset and progression. AMD is driven by a combination of genetic predisposition, natural ageing changes and lifestyle factors, such as smoking or nutritional intake. The mechanism by which these risk factors interact and converge towards AMD are not fully understood and therefore drug discovery is challenging, where no therapeutic attempt has been fully effective thus far. Genetic and molecular studies have identified the complement system as an important player in AMD. Indeed, many of the genetic risk variants cluster in genes of the alternative pathway of the complement system and complement activation products are elevated in AMD patients. Nevertheless, attempts in treating AMD via complement regulators have not yet been successful, suggesting a level of complexity that could not be predicted only from a genetic point of view. In this review, we will explore the role of complement system in AMD development and in the main molecular and cellular features of AMD, including complement activation itself, inflammation, ECM stability, energy metabolism and oxidative stress.

## Age-related macular degeneration (AMD) as a multifactorial disease

AMD is a complex, multi-factorial disease of the elderly. Although genetic studies have been quite successful in identifying genes and processes underlying AMD risk, the understanding of how these genetic variants drive AMD progression is still largely incomplete [1]. As of today, there are strong reasons to believe that the complement system plays a central role in AMD pathogenesis and over activation of the alternative complement pathway is one of the main drivers for disease. Indeed, genetic and epidemiologic studies have been able to pinpoint more than 35 genetic variants conferring risk for developing AMD, many of them mapping to the complement system [2]. Consequently, a number of therapeutic pipelines and clinical trials are currently focusing on regulating the complement system as a therapeutic strategy to treat AMD. However, AMD remains an incurable disease and despite great progress in uncovering the genetic links of AMD, treatment has remained symptomatic. This is, at least in part, due to the fact that AMD is not entirely a genetic disease and indeed, epidemiology studies pinpoint other environmental and lifestyle factors that define the individual risk for disease. In the recent years, a plethora of additional, but just as important, risk factors have been identified, including physiological changes that occur with age and lifestyle, such as smoking and nutrition, which can alter retinal health [3, 4]. At the genetic level, beside inflammation associated with gene variants mapping into the complement pathway, turnover of extracellular matrix (ECM) components and lipid handling are all likely to be important in AMD pathogenesis. At the molecular level, the combination of risk factors result in events defining the disease, including age-related deposition of lipids and protein underneath the retinal pigment epithelial (RPE) cells, metabolic and oxidative stress in RPE cells, changes within the retinal ECM and Bruch’s membrane, complement activation and chronic inflammation [5, 6]. Genetic and environmental risks are likely to converge into critical disease pathways, which may differ in discrete subgroups of patients.

The involvement of different cellular and extracellular components and controllable/uncontrollable risks, makes understanding the generation, pathogenesis and manifestation of a complex disease, such as AMD one of the big challenges in medical research. Subsequently turning this knowledge into prediction, prevention and treatment is yet another imposing task. Capturing the next level of complexity is required in order to understand the interconnection of distinct genetic risks, the interaction with lifestyle factors, the non-response to treatment and the effect of age on the development and progression of the various forms of AMD. This review specifically focusses on the complement pathway as a main driver of disease.

## The complement system

**Fig. 1:**
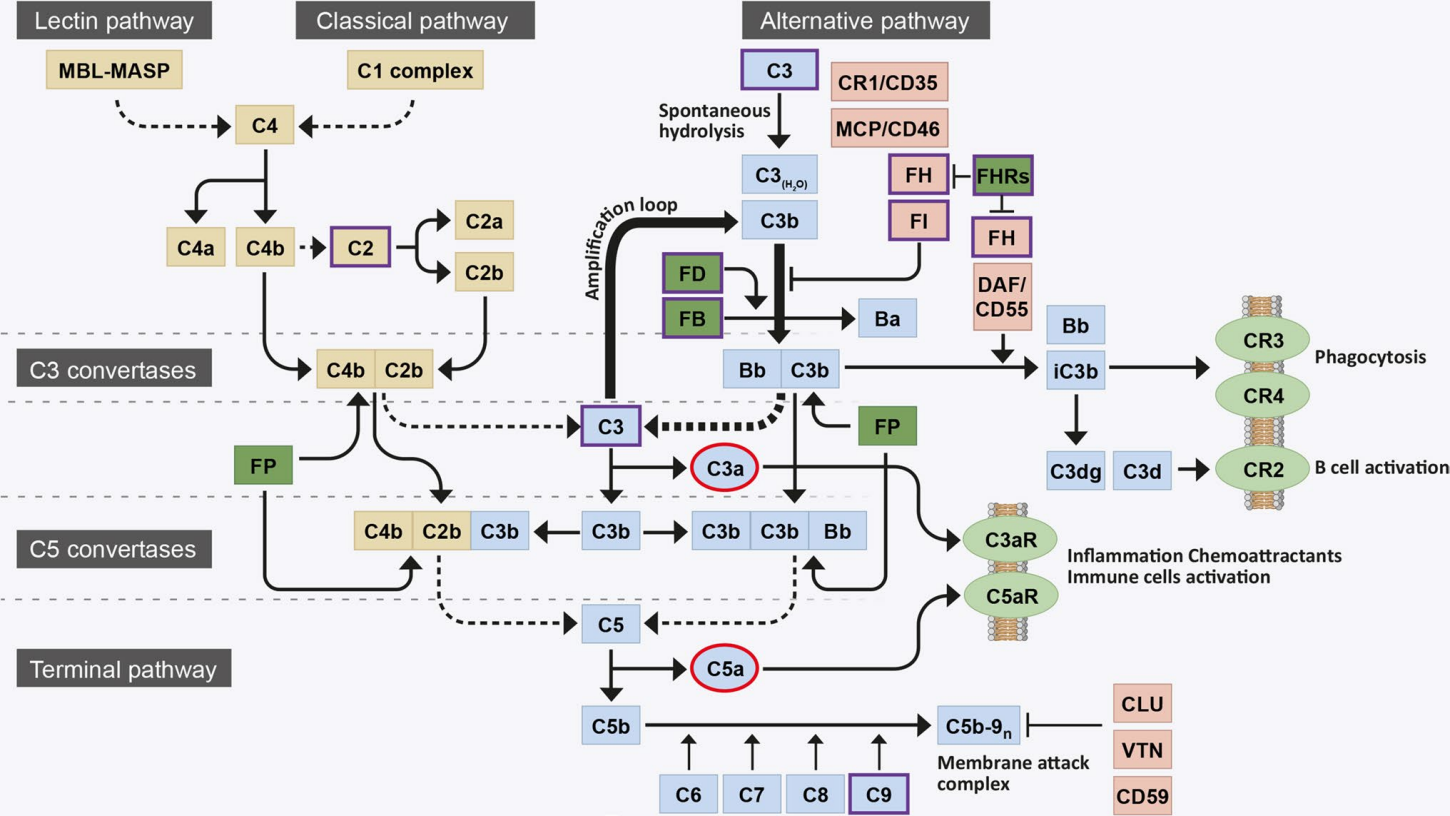
Flow diagram of the complement system activation. The complement system is initiated by three activation pathways: the lectin pathway, the classical pathway (both in yellow) and the alternative pathway (indicated in blue). They all converge in the formation of a C3 convertase, responsible for the breakdown (dotted line) of circulating C3 into C3b. During activation via the alternative pathway, FB binds a spontaneously hydrolysed form of C3 [termed C3(H2O)] and is cleaved by FD to form a distinct C3 convertase, C3bBb. This C3 convertase continuously cleaves C3 into C3b, exposing an internal thioester bond within the protein that allows C3b to become covalently attached to local surfaces. Deposited C3b, a potent opsonin, forms the starting block of a surface bound C3 convertase, that cleaves yet more C3 into C3b and contributes to the amplification loop of complement (thick arrows). C3b is also necessary for the formation of a downstream C5 convertase, responsible for the breakdown (dotted line) of C5 into C5b and the subsequent recruitment of C6, C7, C8 and polyC9 into the C5b-9n complex, also known as the membrane attack complement (MAC). The MAC forms a pore on pathogen/cell surfaces and leads to lysis. Complement system activation is tightly controlled by negative (light red) and positive (dark green) regulators: FI and its cofactors FH, CR1 and MCP promote proteolytic cleavage and inactivation of C3b (iC3b) and FH and DAF promote the disassembly of the C3bBb C3 convertase; CLU, VTN and CD59 inhibit the formation of C5b-9n; FP stabilises C3 and C5 convertase; and the FHR proteins compete with FH for C3b binding and inhibit FI-mediated C3b cleavage. C3b breakdown fragments bind membrane-bound receptors (light green): iC3b binds CR3 and CR4 and mediates phagocytosis; C3dg and C3d bind CR2 and mediate B cell activation. Anaphylatoxins C3a and C5a (highlighted in red), generated with the breakdown of C3 and C5, bind membrane-bound receptors (light green) C3aR and C5aR to promote inflammation and immune cells activation. AMD-associated genetic risk variants mostly occur in the genes of the proteins involved in the alternative pathway of complement (highlighted in violet)

The complement system is a protein cascade composed of more than fifty proteins, which are found in both the fluid phase and bound to cell membranes. The main role of complement is to recognise and mediate the removal of pathogens, debris and dead cells [7]. Proteins of the complement system can be rapidly converted into active forms via a proteolytic cascade triggered by any of the three activating pathways: the classical, lectin and alternative pathways (see Fig. 1) [8, 9]. The classical complement pathway is triggered by the antibody-mediated binding of complement component 1q (C1q) to pathogen surfaces. Activation through the lectin pathway relies on the recognition of pathogen-associated molecular patterns (PAMPs) (d-mannose, *N*-acetyl-d-glucosamine or acetyl groups), on the surface of pathogens or to apoptotic or necrotic cells, by the pattern-recognition molecules mannose-binding lectin (MBL), ficolins and collectins [10]. The alternative pathway of the complement system presents a unique characteristic, since unlike the other initiating pathways, it is constitutively active at low levels due to spontaneous hydrolysis of Complement component 3 (C3) to C3(H_2_0): a process referred to as complement ‘tick-over’.

All three pathways converge in the formation of a protein complex, the C3 convertase, which cleaves C3 into the anaphylatoxin C3a and the central protein in the complement amplification loop, C3b (see Fig. 1). The C3 convertase of the lectin and classical activation pathways is composed of cleaved complement components -4 and -2 (C4bC2b). In the alternative pathway, factor B (FB) can bind C3(H_2_0) and is cleaved by factor D (FD) to form a distinct C3 convertase termed C3_(H2O)_Bb; which, can continuously produce C3b and provide an amplification loop for the complement system activation, independent of the initial trigger. Indeed, C3b is needed to form the downstream C5 convertase, a complex that is responsible for the cleavage of complement component 5 (C5) into the second anaphylatoxin C5a and C5b. Formation of C5b is a prerequisite for the assembly of the membrane attack complex (MAC) composed of C5b, complement components -6 (C6), -7 (C7), -8 (C8) and numerous -9 (polyC9). The function of this complex is to form a pore on a pathogen/hostile material to mediate its cell lysis [8, 9].

The alternative pathway of the complement system needs to be tightly regulated to avoid excessive activation. The serine protease, complement factor I (FI), can cleave C3b deposited on any surface into inactive C3b (iC3b). iC3b cannot contribute to the amplification loop of complement and therefore the action of FI directly both slows down and prevents complement activation. However, FI cannot perform this function alone: it requires the presence of a co-factor protein. There are a number of cell membrane complement regulators, such as: membrane cofactor protein (MCP, CD46), a cofactor for FI; decay-accelerating factor (DAF, CD55), with C3-convertase decay activity; and complement receptor 1 (CR1, CD35), which possesses both cofactor and decay activities [11]. However, the blood borne co-factors complement factor H (FH), and its alternative splice variant factor H-like protein 1 (FHL-1), are the only two complement regulators that will allow FI-mediated C3b cleavage on acellular surfaces, such as ECM. Moreover, FH, by physically displacing FB from C3b, also accelerates the decay of the C3bBb convertase [12]. In addition, other cell-bound regulators inhibit the terminal pathway of complement, by interfering with the formation of MAC on cellular membranes and the subsequent cell lysis, such as clusterin (CLU), vitronectin (VTN) and CD59 [13, 14].

Both FH and FHL-1 are expressed by the single *CFH* gene found in the region of complement activation (RCA) cluster on chromosome 1 [15, 16]. Downstream of the *CFH* gene are five factor H-related genes (*CFHR1-*5) and in contrast to the FH and FHL-1 regulatory proteins, the five FH-related proteins (FHR1-5) are believed to act as positive activators of the complement system [15, 17]. The FHR proteins compete for binding to C3b, but do not themselves share the FI co-factor domain possessed by FH and FHL-1, and therefore actively prevent FI-mediated C3b breakdown, leading to increased complement turnover [17]. As the activated complement system is turning over, the release of the anaphylatoxins C3a and C5a exert specific additional functions. These small peptides act as chemoattractants, which recruit circulating immune cells to the site of complement activation, and by binding to specific cell receptors (i.e., C3aR and C5aR respectively) promote degranulation and release of pro-inflammatory cytokines [18].

Clearly, tight regulation of complement activation is required in order to maintain tissue homeostasis and prevent unnecessary inflammation and tissue damage. Over-activation of the complement system is associated with driving the pathogenesis of a number of systemic and organ specific, diseases. Most recently, a strong genetic and biochemical link have been made between poor regulation of complement activation in the back of the eye and the blinding disease AMD.

## AMD pathophysiology

**Fig. 2:**
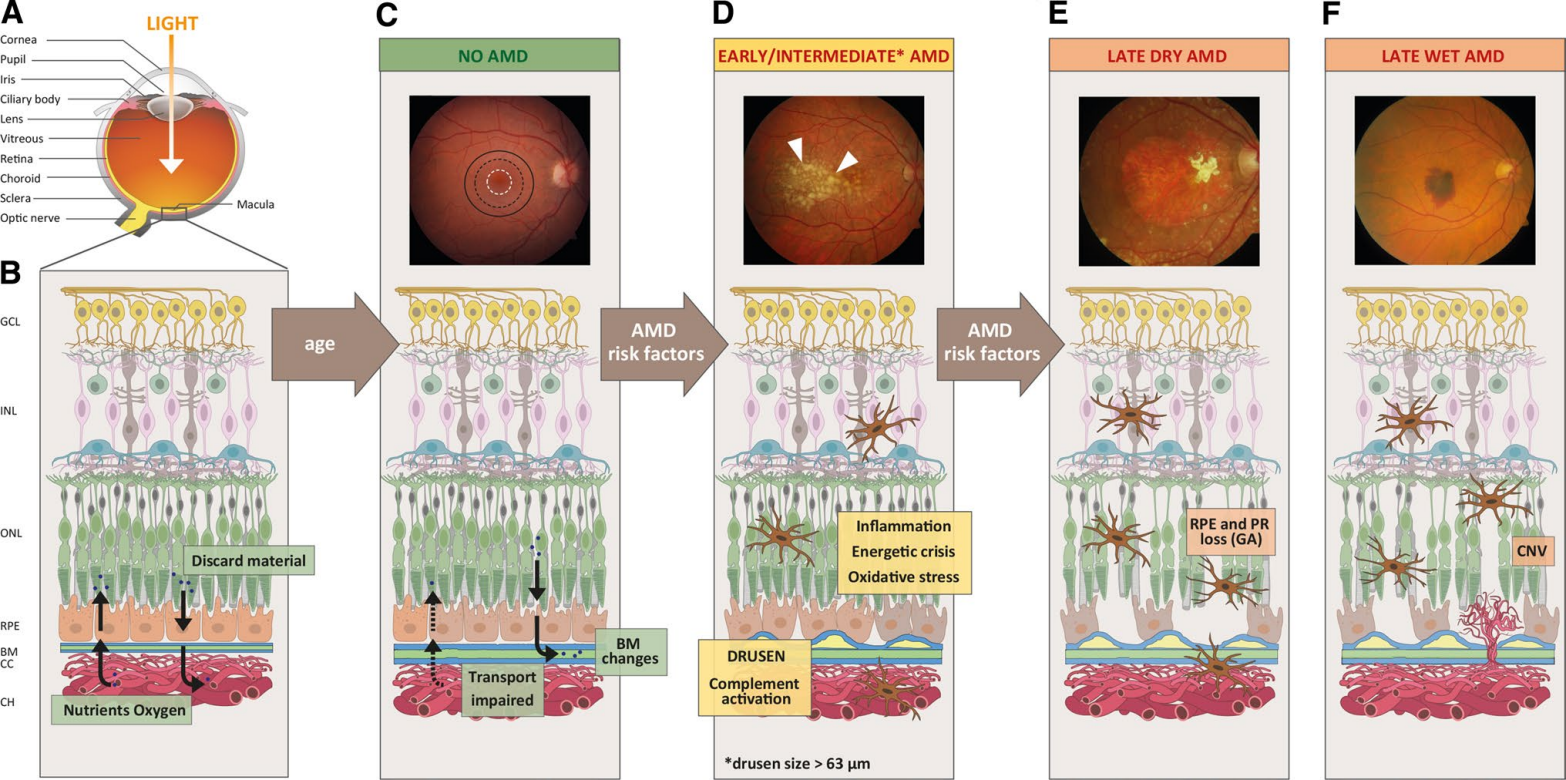
Schematic of the human eye in health, age and AMD. **a** Diagram highlighting the anatomical features of the human eye. **b** Schematic of a healthy human retina with its cell layers and transport of nutrients across Bruch’s membrane, where; *GCL* ganglion cell layer, *INL* inner nuclear layer, *ONL* outer nuclear layer, *RPE* retinal pigmented epithelium, *BM* Bruch’s membrane, *CC* choriocapillaris, and *CH* choroid. **c–f** Progression of AMD shown by fundus images and schematic changes within the retinal cell layers. In older patients without AMD **c**, fundus imaging is normal and the macula (black circle), parafovea (black dotted circle) and fovea (white dotted circle) appear intact. On a structural level, BM is altered and transport properties are becoming impaired. Additional AMD risk factors **d** promote inflammation, oxidative stress, energetic crisis, complement activation and drusen formation. Drusen can be visualised in fundus images as yellow spots (white arrows). Progression to later stages of AMD can lead to **e** geographic atrophy (GA) characterised by defined areas of RPE cell death (termed dry AMD) and **f** choroidal neovascularisation (CNV) into the retina (termed wet AMD), ultimately leading to photoreceptors (PR) cell death. Fundus images were obtained from the Macula Reading Centre, University of Tübingen (UKT)

Age-related macular degeneration (AMD) is a progressive degenerative disease of the macula, the central region of the retina, which ultimately leads to blindness. AMD poses a great burden for society worldwide, due to the debilitating features of the disease and an ever-increasing number of cases as the world’s elderly population expands. Indeed, 8.7% of the world’s population is affected by AMD, with disease prevalence rising to 12.3% in Europeans [19]. Since the disease manifests itself in individuals over 60 years of age, it is estimated that 300 million people will be affected by 2040, driven primarily by increasing life expectancy and the increasing number of the elderly population worldwide [19]. AMD patients lose the central field of vision and vision acuity, making it impossible to read, write, drive and recognise faces, all of which greatly impede an independent and active life [20]. Clinically, AMD progression is sub-divided into early, intermediate and late stages of the disease (see Fig. 2). Early stages, which are often asymptomatic, are characterised by the presence of sub-retinal deposits, called drusen, in the macula region, which typically measure 63 μm in diameter [21]. The increase in drusen size, number and changes in their pigmentation, which can be detected in fundus imaging of the retina, occur in the intermediate symptomatic stages of the disease [22]. Late-stage AMD is divided into two forms, but both end in significant visual impairment. Late AMD can affect the retina either as a ‘wet’ form, characterised by unorganised choroidal neovascularisation (CNV) or a ‘dry’ form, characterised by defined islands of RPE cell death, referred to as geographic atrophy (GA).

Despite recent advances in the treatment of CNV for wet AMD with anti-vascular endothelial growth factor A (VEGF) agents [23], anti-VEGF treatments remain ineffective for some patients and a significant proportion of these patients still develop severe visual loss and progress to legal blindness over time [24]. Recent evidences point to the possibility of an association between anti-VEGF therapy and progression of geographic atrophy [25]. There is currently no satisfactory treatment for the dry form of AMD, although clinical trials have investigated an increasing number of therapeutic options [26] (Table 1). Geographic atrophy generally starts with atrophy of the RPE and choriocapillaris adjacent to the fovea, and foveal function and central visual acuity is initially spared. Cessation of lesion growth in GA would prohibit major visual loss. In this case, the only potential option to slow down the progression of the disease is nutritional supplementation (AREDS formulation) [27, 28].

**Table 1:** Recent complement mediating therapeutics directed against AMD in clinical trials

Therapeutic (alt. name)^*a*^	Drug class	Delivery method	Complement target	Company	AMD form targeted	Clinical trial
*APL-2*	Peptide	Monthly intravitreal injection	C3	Apellis	Dry	NCT03525600 NCT03525613 Phase III
*Zimura* (ARC1905)	Aptamer-based inhibitor	Monthly intravitreal injection	C5	IVERIC bio	Dry	NCT04435366 Phase III
					Wet	NCT03362190 Phase II
*IONIS-FB-LRx*	siRNA	Subcutaneous injection	FB	Ionis Pharmaceuticals	Dry	NCT03815825 Phase II
*GEM103*	Biologic	Monthly intravitreal injection	FH	Gemini Therapeutics	Dry	NCT04246866 Phase I
*GT005*	AAV gene therapy	Single sub-retinal injection	FI	Gyroscope Therapeutics	Dry	NCT03846193 NCT04437368 Phase I
*AAVCAGsCD CD59*	AAV gene therapy	Single sub-retinal injection	CD59	Hemera Biosciences	Dry	NCT03144999 Phase I
**Recently terminated trials**						
*Lampalizumab (FCD4514S)*	Antibody Fab fragment	Monthly intravitreal injection	Factor D	Genentech/Roche	Dry	NCT02247479 NCT02247531 Phase III Terminated
*CLG561 (in combination with LFG316)*	Monoclonal antibody	Monthly intravitreal injection	Properdin	Alcon/Novartis	Dry	NCT02515942 Phase II Terminated

^*a*^In some instances, the therapeutics listed have been known previously by a different name

### The macula

The retina, located in the posterior part of the eye, is a multi-layered tissue that is divided into: the neurosensory retina, which consists of photoreceptors cells (rods and cones) and a network of neurons and specialised glial cells; and the RPE cells, which are located on a thin ECM called Bruch’s membrane (Fig. 2). Nutrients from the blood are supplied to the retina via a dual vascular system: retinal vasculature within the neurosensory retina itself; and the choriocapillaris that comprises a network of fenestrated blood capillaries that reside under Bruch’s membrane (Fig. 2). The central region of the retina is referred to as the macula, which measures 5 mm in diameter and whose central region (termed the fovea) is comprised of the highest density of cone photoreceptors in the eye and confers central visual acuity. The fovea is surrounded by a rod-dominated area termed the parafovea [29]. The cells of the macula, and particularly the fovea, mainly rely on the blood supplied from the choroidal side of the blood/retinal barrier, since the neurosensory vascular system is limited in the macular area to allow a better light absorption from the cones and a more detailed and defined vision [30].

The RPE cells are of vital importance for the preservation of retinal homeostasis, especially in the macula region where the photoreceptors are highly condensed. Indeed, RPE cells are polarised epithelial cells distributed in a monolayer between Bruch’s membrane and the photoreceptors. In this way, RPE cells regulate the uptake of nutrients and oxygen from the choroid to the photoreceptors and control the release of waste material into the general circulation at the Bruch’s membrane site [31]. Moreover, RPE cells are responsible for the turnover by phagocytosis of photoreceptor outer segments (POS) that are shed as part of the necessary recycling of visual pigments: a process that supports a correct visual cycle [32]. Although each RPE cell serves approximately 20–30 photoreceptor cells (both rods and cones), this number varies across the retina and recently, it has been suggested that RPE cells are in closer contact to rods compared with cones photoreceptors [33]. The RPE cell monolayer also provides other essential functions such as providing structural support, contributes to the production and secretion of a variety of important growth factors, and perhaps most importantly protects the retina from oxidative stress thanks to the presence of melanosomes which absorb excess light this might otherwise contribute to considerable oxidative stress [5]. Moreover, oxygen consumption in the retina is incredibly high, needed by both RPE cells and photoreceptor cells, making the macula the most metabolically active region in the body [34].

Finally, the RPE cell layer and the choroid are separated by the acellular Bruch’s membrane. This ECM is composed of five separate layers: the basement membrane derived from RPE cells, an inner collagenous layer (ICL), the central elastic layer (EL) and outer collagenous layer (OCL) and the basement membrane on the choroidal side derived by the endothelial cells of the choriocapillaris [35]. The main component of Bruch’s membrane are collagens, fibronectin, laminin, heparan sulphate proteoglycans (HSPGs) and dermatan sulphate proteoglycans (DSPGs). Bruch’s membrane not only acts as a physical support for RPE adhesion [36], but also contributes directly to the selectivity of the outer blood/retinal barrier by dictating which proteins can, and can-not, diffuse through [37].Therefore, any change to the choroid/Bruch’s membrane/RPE interface, whether through disease, environmental effect or age, will change the nature of these interactions and may affect the natural homeostasis of the retina and support of normal vision.

## Risk factors for AMD

AMD is caused by a combination of risk factors, which together define an individual’s predisposition to AMD, and include ageing, environmental and lifestyle risk factors and genetic predisposition. In the following sections we will describe potential scenarios on how those risk factors, complement system and cellular processes interact and contribute to AMD onset and progression.

### Age-related changes

The main predisposition for AMD is provided by the physiological changes that occur in the retina with the advancement of age. RPE cells are subjected to age-related alterations, including pigmentary changes, the reduction in melanosomes and an increase in lipofuscin [38]. Moreover, with increasing age, and more noticeable in AMD patients, there is a decrease in the size and number of mitochondria within the RPE cells, as well as a loss of cristae and reduction in mitochondrial matrix density [39]. These changes alter RPE energy metabolism and may render RPE cells more vulnerable to photo-oxidative stress and oxidative damage and therefore unable to protect the retina. Indeed, mitochondria damage induced by superoxide dismutase 2 (SOD2) knock-out leads to retinal damage in aged mice similar to an AMD phenotype [40]. RPE cells are also sensitive to the changes in their underlying ECM (i.e., Bruch’s membrane) and for this reason the age-related changes in the choroid/Bruch’s membrane with advancing age are also fundamental contributors to AMD pathology.

Both Bruch’s membrane and the choroid undergo modifications due to ageing that may be deleterious to the homeostasis of the RPE, and subsequently the photoreceptor cells. Bruch’s membrane becomes thicker with age caused by the increased deposition, and subsequent crosslinking of collagens fibers resulting in a decrease of its permeability [35]. Another age-related change in Bruch’s membrane is a significant reduction in the amount of HSPGs, which are important for RPE adhesion and a key anchoring partner for soluble regulators of complement activation (see Sect. “The complement system and ECM” below) [41]. Moreover, in aged Bruch’s membrane there is an accumulation of advanced glycation end products (AGEs), which consist of glycated and oxidised proteins and lipids [36] and can promote inflammation via activation of the AGE receptor (RAGE) on RPE cells and immune cells [42]. Indeed, a number of leucocytes have been found to accumulate in the choroid of aged retinas, including the recruitment and activation of macrophages and the degranulation of mast cells, leading to an environment of chronic inflammation in AMD patients [43, 44].

Similarly, the choroid itself is also affected by age. The choroid possesses good plasticity properties, allowing rapid physiological changes when needed, but the aged choroid loses flexibility and becomes thinner [45]. This leads to a reduction in blood flow and nutrient/oxygen supply to the retina [46], leading to starvation and hypoxia and forcing the RPE cells into an extreme level of metabolic effort. The culmination of these Bruch’s membrane and choroidal modifications weakens the capability of the choroid to deal with the discarded material from the retina and RPE cells, since diffusion and transport of such materials across Bruch’s membrane are impaired. Therefore, waste material from RPE cells continue to accumulate and it is believed to mainly consist of oxidised lipids from POS recycling [47]. The more debris accumulate, the more Bruch’s membrane properties are impaired, initiating a vicious circle of continuous debris deposition, which are believed to ultimately culminate in drusen formation. The exact drusen composition is not fully clarified and varies among donors and disease stage. However, beside the oxidised lipids component, several proteins have been found in drusen including complement system and inflammatory factors, such as vitronectin, Serum amyloid P component, apolipoprotein E (APOE), immunoglobulin light chains and complement proteins (C5 and the C5b‐9 complex) [48].

Drusen are morphologically divided in hard and soft drusen, which can be detected as yellow-white spots in fundus imaging of the retina (see Fig. 2d, e). Hard drusen measure less than 63 µm and are characterised by sharp, defined edges, while soft drusen are larger and with a much less well-defined shape [22]. Drusen are often present in small numbers in any aged eye, but they increase in AMD patients. This implies that other mechanisms underlie AMD pathogenesis beyond the simple presence of drusen themselves.

Furthermore, additional types of deposits accumulate in the retina, for example basal laminar deposits (BlamD) and basal linear deposits (BlinD), both appearing flat and continuous underneath the RPE cells. Basal laminar deposits are composed of ECM material between RPE cells and their basal membrane, while basal linear deposits, consisting mainly of lipids, are located more internally in the Bruch’s membrane. BlinD are situated in the same location of drusen and are strongly associated with early AMD [49].

### Environmental risk factors

The aged retina is believed to be more susceptible to stress stimuli and therefore environmental external factors that increase stress are likely to be detrimental, while healthy life-style habits are considered beneficial (reviewed in [50]). Following age, the next most important environmental risk factor for AMD is smoking, which is associated with a 2–fourfold increase risk for any type of AMD [51]. The impact of cigarette smoke on AMD risk is dose-dependent and, indeed, after quitting smoking the risk of developing AMD decreases. Moreover, twenty years after stopping smoking the risk of AMD is considered to be minimal [52]. The main biological consequences attributed to smoking is the increase in oxidative stress and inflammation in the retina and changes within the choroid, such as vasoconstriction and vessels branching [53]. The role of metal ions associated with smoking and their increased blood levels [54] and their accumulation in tissues has been implicated in AMD, and interestingly, smoking-related changes in the levels of metal ions associated with cigarette smoke are also positively associated with cataract formation [55].

An individual’s diet can alter an individual’s risk of AMD, depending on their dietary regime. The ‘Mediterranean diet’ reduces the risk of developing AMD [56, 57]. Many of the classical components of this type of diet possess antioxidants properties and have been shown to be protective in independent studies. For example, the Mediterranean diet is rich in fruits and vegetables, which contain vitamins and carotenoids, also protecting from oxidative stress [58]. Moreover, fish intake is shown to be protective in AMD, probably due to the high content of poly-unsaturated fatty acids, eicosapentaenoic acid (EPA) and docosahexaenoic acid (DHA) [59]. Conversely, the ‘fast food diet’, i.e., hyperglycaemic diet rich in carbohydrates and sugar beverages, represents a significant risk factor for AMD [60, 61]. Indeed, mice fed with a high fat diet, cholesterol, and fructose-supplemented water, showed signs of retinal degeneration similar to AMD including basal deposits, RPE cells loss and Bruch’s membrane thickening [62].

### Genetic risk

Genome-wide association analyses (GWAS) have by far been the most successful in creating a genetic risk map for the single nucleotide polymorphism (SNP) associated with AMD [63]. The first risk SNP (rs1061170) associated with a strong effect on AMD was a common polymorphism (p.Tyr402His) in the gene for complement factor H (*CFH*) published in 2005 [64]. The *CFH* gene is located on chromosome 1q31, a locus that had previously been identified by linkage analyses in a large family affected by AMD [65]. The second strong risk locus identified by GWAS was reported a year later on 10q26 [66], which turned out to be associated with an open reading frame that harbours the gene for age-related maculopathy susceptibility 2 (*ARMS2*), an ECM protein of unknown function [67] and HtrA serine peptidase 1 (*HTRA1*), a serine protease processing ECM proteins [68, 69]. Polymorphisms in the *CFH* and *ARMS2/HTRA1* genes account for an important proportion of the AMD risk. Genetic risks in ARMS2 and the complement pathway are present in the majority of late AMD cases, but are mostly combined with risks in other pathways [70].

Since then, GWAS studies have identified another 33 discrete loci with more than 50 independently associated genetic variants [71]. Most of these genetic risk factors have been confirmed in independent studies [70, 72]. Although polymorphisms in the *CFH* and *ARMS2/HTRA1* genes bear the highest single attributable risk scores for a single risk allele, additional genetic variants have been associated to AMD: in or near genes of the complement system (*CFB, CFI, C2, C3*); genes involved in ECM remodelling as Collagen Type VIII Alpha 1 Chain (*COL8A1*) and Tissue Inhibitor of Metalloproteinases 3 (*TIMP3*); genes involved in cholesterol metabolism as ATP-binding cassette transporter (*ABCA1*), Apolipoprotein E (*APOE*), Cholesteryl ester transfer protein (*CETP*), Lipase C, Hepatic Type (*LIPC*) and genes in less well-defined pathways, e.g., Rho GTPase Activating Protein 21 (*ARHGAP21*) and Beta 3-Glucosyltransferase (*B3GALTL*) [73]. The odds ratios of the representative SNPs in these genes typically fall into the range of 1.1–3.0, with a majority < 2; thus, each locus only has small to moderate contribution to the risk of the disease. In contrast, risk variants in *CFH*, *ARMS2/HTRA1, C2/CFB/SKIV2L* and *C3* determine the majority of the genetic risk of AMD. The discovery of rare genetic variants, some with large effect sizes, have not added any more risk genes to the overall genetic risk profile for AMD but simply confirmed the role of these genes in AMD pathogenesis. Sequencing of candidate genes in case–control studies and in AMD families resulted in the identification of rare variants in known AMD loci, i.e., in the genes *CFH*, *CFI*, *C3,* *C9*, in addition to non-complement-related genes [74].

These genetic association studies have led to the identification of the alternative complement pathway as a main driver of disease and have recently turned into useful tools for the validation of associated candidate therapeutic targets as well as providing novel biomarkers for further stratification of disease risks. The discovery of five AMD risk alleles coding for proteins of the alternative complement pathway highlights the role of the innate immune system in the development of AMD.

Copy number variations in multi-allelic gene loci harbouring complement genes may increase or reduce the risk to developing AMD, especially in the late stage of disease and at a greater age [75]. A study assessing copy number variations in the complement gene *C4*, which has so far not been associated with an attributable risk to AMD, suggests statistical significance for a protective association of increasing copy numbers of one *C4* gene transcript (*C4A*) and AMD. Conversely, *C4A* copy numbers were lower on a risk haplotype characterised by the presence of the SNP rs204993, but higher on a protective haplotype characterised by the rs429608 SNP [75].

Increasing evidence suggests, that the drivers and risks for early AMD may not be totally identical with those responsible for progression into the late stage of disease. This is likely for both genetic as well as non-genetic risks [57] that may negatively or positively influence different phases of disease manifestation. A recent GWAS study on the genetics of early AMD identified a variant near the CD46 gene to be more or less exclusively associated with early AMD and not late AMD. Besides CD46, this locus also harbours other complement genes (such as CR1 and CR2). The results from this study seem to indicate that the genetics that governs both, early and late AMD is complement mediated while the impact of specific variants associated with early or late AMD may be discrete [72]. In line with this observation is the finding that a risk variant in C3 (rs2230199) counterintuitively associates with slower growth rates in GA [76], whereas the same risk allele correlates with an increased overall risk to develop late stage AMD [77].

Ironically, the *CFH* locus is actually the second most associated risk locus for AMD. The strongest risk variant, however (dbSNP ID: rs10490924) resides in the *ARMS2/HTRA1* risk locus whose biochemical pathways remain poorly characterised [67]. Given our better understanding of the proteins arising from the *CFH* risk locus, it is perhaps unsurprising that this has become the focus for therapeutic intervention. ARMS2 was found to be a small extracellular matrix ECM protein of about 11 kDa [67] secreted in an atypical fashion by secretory autophagy [78]. ARMS2 risk variants are strongly associated to all stages of AMD. Moreover, a significant gene–environment interaction with cigarette smoking was confirmed [79]. Besides binding ECM components that reside in the elastic layer of Bruch’s membrane [67], a recent study has found evidence that human monocytes, as well as a subfraction of retinal microglia cells, express ARMS2 on their surface [80]. Deficiency of the ARMS2 protein was observed in monocytes homozygous for the AMD associated genetic polymorphism rs10490924 in the ARMS2 gene. The study suggests that ARMS2 loss of expression or loss of function may promote AMD. The binding of ARMS2 to the complement activator properdin suggests that the function of ARMS2 may be linked to complement regulation, and strengthens the role of local complement dysregulation in both the neuroretina as well as in the interface of Bruch’s membrane and choriocapillaris as a major risk for AMD.

## The role of complement in AMD pathogenesis

**Fig. 3:**
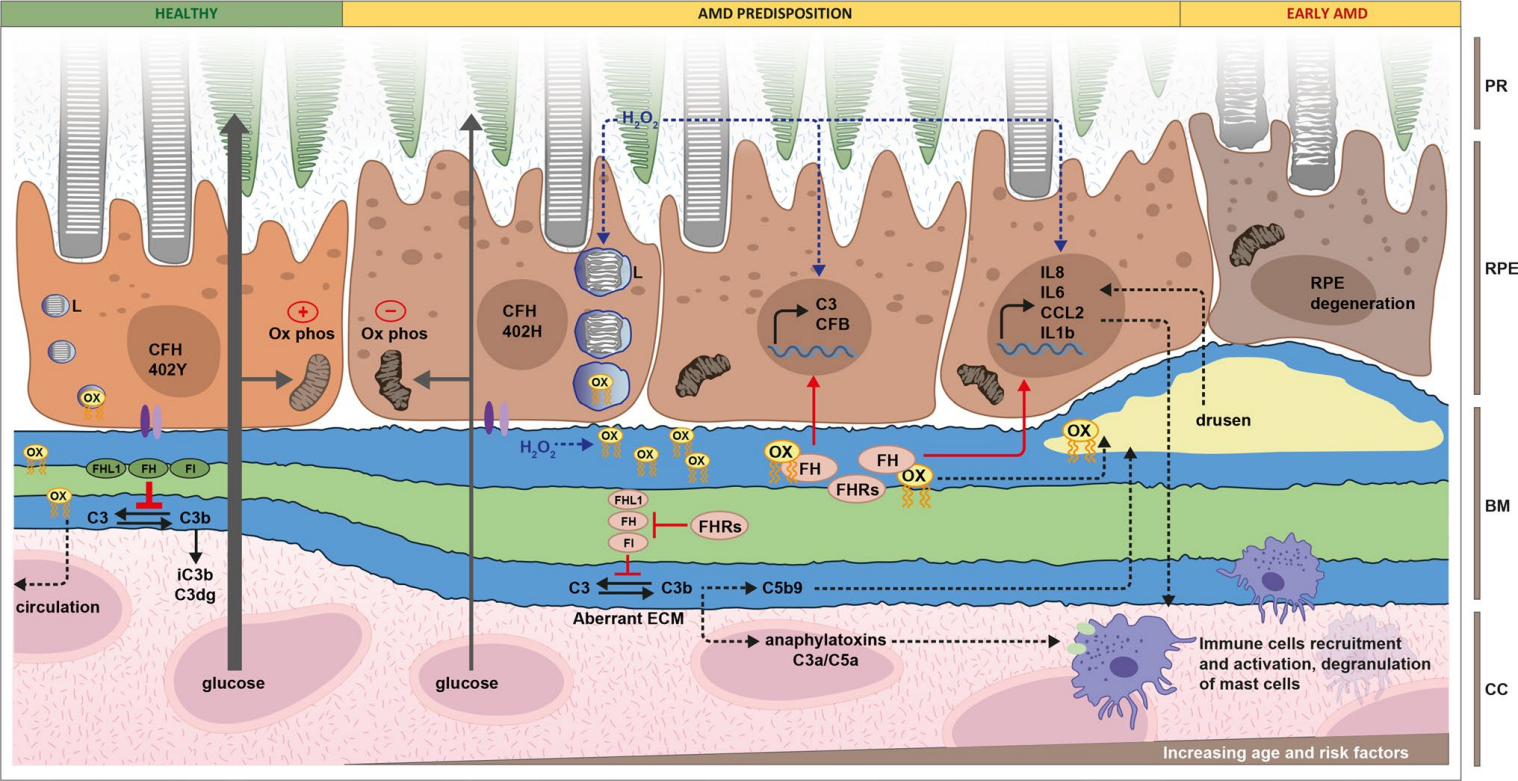
Involvement of complement system in AMD predisposition. In healthy conditions, RPE cells, exposed to healthy Bruch’s membrane (BM) and carrying the FH 402Y polymorphism, transport efficiently glucose from the Choroid/choriocapillaris (CC) through BM to the photoreceptors (PR), phagocyte POS and eliminate oxidised lipids (ox) into the circulation. Integrins (violet) anchor the RPE cells to BM, FI and cofactors FH and FHL1 (green) inhibit complement activation and mitochondria respiration (ox phos) is intact. AMD predisposition is provided by the combination of genetic risk, increasing age and external risk factors. RPE cells carrying the AMD-associated FH 402H polymorphism show reduced mitochondrial function, activity of phagolysosomes (L) and integrin interactions with BM. With increasing age and external risk factors, the BM ECM becomes even more altered and glucose transport is impaired. At this stage complement turnover is stimulated by aberrant ECM and a lack of effective inhibition due to the presence of high-risk variants in FI, FH, FHL1 (red) and the accumulation of FH antagonists, such as the FHR proteins (red). Products of complement turnover accumulate in the intercapillary septa and within BM itself. Additionally, oxidative stress is increased, which provides metabolic stress to the RPE cells and can stimulate inflammation. Hydrogen peroxide (H_2_O_2_) and products of lipid peroxidation (ox, yellow) accumulate in the altered BM, promote the upregulation of inflammatory cytokines and the high-risk FH 402H variant and FHR accumulation amplify this effect. In early AMD, continuous accumulation of oxidised lipids, complement activation products and MAC accumulation in drusen further promotes inflammation. Local inflammation, together with the release of anaphylatoxins C3a and C5a, causes the recruitment and activation of immune cells and degranulation of mast cells into the BM/RPE/retina interface, aggravating retina homeostasis: RPE cells degenerate and are unable to support rod photoreceptor cells, which start to show signs of damage

Here, we will explore possible scenarios in which the complement system contributes to molecular mechanistic drivers of AMD. Crucial steps in AMD pathobiology, believed to be associated with complement dysregulation, are summarised in Fig. 3.

### Activation of the alternative pathway

Dysregulation of the complement system is a significant driver of AMD pathogenesis (see Fig. 3). Initially, early studies debated whether systemic complement versus local complement activity contributed to disease in the eye. Today, however, it is generally accepted that the complement-mediated molecular mechanisms driving AMD result from a mixture of both locally synthesised complement proteins (e.g., FD, FHL-1), as well as systemic complement proteins that are conferring an effect locally in tissues (e.g., FHRs). More recently, several complement system activation products have been found elevated systemically in AMD patients [81] and complement turnover, assessed via the C3d/C3 ratio, proved a correlation between systemic complement activation levels and disease stage [81]. Indeed, patients with intermediate AMD and late dry AMD (with central GA or inactive CNV) show higher levels of complement activation compared to both controls and early AMD group. Interestingly, in patients with active CNV the levels of complement activation were relatively low. The differences in complement turnover were more pronounced in patients with adverse *CFH* genotype [81]. In terms of local complement activation, significantly elevated C3b deposition has been observed in donor eyes which carry genetic risk at chromosome 1 (around the *CFH* gene) even before the manifestation of the disease itself [82], implying that poorly controlled complement turnover in the ECM of the choriocapillaris occurs much earlier in the disease process than previously thought.

Conversely, products of complement system activation have been found accumulating in the choroid even very early in life and in donors with no discernible genetic risk for AMD [83]. Accumulation of terminal pathway MAC were found to be present in the histological sections of eyes from young donors in their as early as 21 years of age. It has been suggested that a constant low-grade turnover of complement is required to maintain normal tissue homeostasis in the eye and that low quantity of MAC may be necessary to help with the clearance of POS photoreceptors collected by RPE cells every day. With advancing age and in pathological situations like AMD, MAC levels increase, and again MAC formation has been reported mainly in the choriocapillaris and capillary septa. In late stages of AMD, MAC localises also in proximity of RPE cells, suggesting some sort of tolerance of RPE cells versus complement-mediated injury, at least in the early stages of disease [82–84].

Despite RPE cells producing C3 and all the proteins required for complement tick-over [85], it remains unclear whether RPE cells themselves can produce all the necessary components required for the formation of the terminal pathway MAC. Anderson et al. found that the predominant source of the components of the terminal pathway, except for C5 and C7, was from the choroid and not RPE cells [86]. This adds weight to the hypothesis that the RPE cells are specifically designed to resist complement-mediated cell death: RPE cells are incredibly difficult to kill with complement in vitro unless the cells are put under significant amount of stress. Conversely, a single-cell transcriptomic analyses of retinal tissues found the expression of all factors of the terminal pathway in RPE cells [87].

### The complement system and ECM

Changes in the composition of Bruch’s membrane and choriocapillaris ECM have been observed in the very early stages of AMD, which can be followed by BlamD and drusen formation. Naturally occurring age-related changes to this ECM are well documented (see Sect. “Age-related changes”) and it is likely that these changes gradually create an environment where underlying genetic risk is exposed. For example, the only blood borne FI co-factors, FH and its truncated version FHL-1 (itself shown to be more prominent in Bruch’s membrane [88]), are solely responsible for protecting ECM from C3b deposition, complement turnover, and inflammation. FH and FHL-1 anchor to ECM through recognising and binding sulphated glycosaminoglycans (GAGs), such as HSPG and allow FI to inactivate any C3b that may become deposited. The common FH/FHL-1 Y402H polymorphism, which is believed to confer ~ 50% of attributable risk for developing AMD [89, 90], alters the binding site in both proteins for these sulphated GAGs [91], where the 402H disease-associated variant being more sensitive to changes in sulphation [92]. The observed age-related loss of HSPG in Bruch’s membrane (up to 50% from 30 to 70 years of age [41]) represents an age-related loss of anchoring ligands for FH and FHL-1. It is possible that in young ECM, awash with potential FH/FHL-1 anchoring partners, the Y402H polymorphism is well tolerated, and that the age-related decrease in these GAGs leads to less FH/FHL-1 binding and an increase in complement turnover and immune cell recruitment into the choroidal space: both hallmark features of AMD. This would imply then that, a) patients homozygous for the FH 402H variant would carry a far greater risk from AMD in old age than individuals who were heterozygous for the 402H variant, which is indeed the case (7.4-fold greater risk opposed to 2.7-fold [64]) and, b) correct genetic stratification of patients is required for successful clinical trials (see Sect. “Complement-mediated therapeutic strategies for treating AMD” below). Furthermore, rare mutations in the *CFH* locus that lead to haploinsufficiency of FHL-1, which has been associated with Early Onset Macular Drusen (EOMD), a disease with striking similarity to AMD but occurs in a patients’ twenties or thirties [93].

Although changes in ECM may well lead to complement activation, it also has profound effects on the gene expression and protein secretion profiles of the RPE cells themselves. A recent study has identified that RPE cells, through their α5β1 integrin receptor, recognise and bind to the ‘RGD’ binding motif in FHL-1 when the protein is immobilised onto a surface [94]. This binding interaction leads to an increase in the expression of heat-shock protein genes and changes in the EIF2 signalling pathway and mineralocorticoid receptor signalling. Ultimately it was shown that RPE cells bound to FHL-1 were protected from oxidative stress-induced cell death, even when compared to the same cells bound to fibronectin. Less FHL-1 in Bruch’s membrane would not only lead to increased complement turnover, but also destabilise the adjacent RPE cells and alter their ability to deal with oxidative stress (see Sect. “Complement system and oxidative stress in AMD” below).

It is not only natural ECM changes that can lead to complement activation. It has been shown indeed that RPE cells mutated for EFEMP1, gene coding for Fibulin-3, deposited aberrant ECM, which induces C3 turnover in naïve RPE cells [95] (see Fig. 3). In addition, iPSC-derived RPE cells carrying AMD-associated mutations in EFEMP1 or TIMP3 genes, generate more and larger drusen-like deposits compared to controls and increase expression of complement proteins, including C3 and C5 [96]. Moreover, FH directly binds Fibulin-3 and the FH 402H variant colocalise with Fibulin-3 in soft drusen of AMD patients, indicating that this interaction may be involved in drusen formation [97]. Another modification which occurs in aged Bruch’s membrane is the accumulation of nitrite groups and nitrite-modified ECM affects attachment, increase the expression of complement genes and C3a accumulation in iPSC-derived RPE cells from AMD patients [98, 99]. Bruch´s membrane acts as a filter for complement protein passive diffusion between choroidal site and retinal side, independent of the site of production. In AMD, the accumulation of lipids alters the permeability of Bruch’s membrane. Indeed, while under normal conditions FHL-1, FD and C5a diffuse freely, the complement regulator FHL-1 does not diffuse as efficiently through Bruch´s membrane isolated from AMD patients compared to controls, but the diffusion of FD, which supports complement activation, remains unhindered [37].

### Complement system and inflammation

Another striking feature of AMD is the presence of chronic inflammation in the eye. By definition, the most obvious consequence of complement activation, independently from the source of complement proteins, is the recruitment and activation of immune cells mediated by complement activation in local tissues and the release of the anaphylatoxins C3a and C5a (Fig. 3). The immune cells involved in AMD comprise not only retinal resident microglia cells, but also circulating lymphocytes and monocytes/macrophages and mast cells [43, 44]. Indeed, the stimulation of monocytes by C3a can lead to interleukin 1 beta (IL-1β) secretion and NLRP3 inflammasome activation [100], and both C3a and C5a cause an increase in NF-kB signalling in monocyte derived dendritic cells [101]. Interestingly, mast cell degranulation by C3a and C5a [102] releases the proteases tryptase and chymase, which are known to confer ECM degradation and remodelling (see Sect. “The complement system and ECM” above) [103, 104]. Resident microglia are also affected by complement dysregulation. Indeed, uncontrolled C3 activation in *Cfh*^*−/−*^ knock-out mice, and the complete absence of C3 in *C3*^*−/−*^ knock-out mice, both negatively affect aged retinas [105]. Both in the absence of either FH or C3, mice showed reduced number of activated microglia (Iba1 positive cells) and the *C3*^*−/−*^ mice also saw an increase in tumour necrosis factor (TNFa) expression [105], highlighting the importance of balanced complement activation for retinal heath. It has also been noted that resident microglia cells overexpress C3, when retinas are exposed and stressed with continuous bright light [106].

Classical immune cells aside, complement activation can also stimulate the nearby RPE cells into secreting a range of inflammatory cytokines, such as interleukin 6 (IL-6), interleukin 8 (IL-8) and Monocyte Chemotactic Protein 1 (CCL2) [107] (Fig. 3). Moreover, microglia cells also modulate the activity of RPE cells, providing even more cellular feedback in his tightly knitted tissue. Indeed, blockage of Adenosine A2a Receptor (A2aR) in microglia cells inhibits the inflammatory response, inflammasome and complement activation in ARPE19 cells [108], highlighting how complex the interactions are among all the cell types represent within the retina. To make it even more complex, the photoreceptors themselves can mediate inflammatory responses in the retina. In mice models of retinal degeneration, at the periphery of atrophic lesions, the retina is exposed to the systemic circulation due to outer blood–retinal barrier breakdown. In these circumstances, POS acts as a complement activator through the classical and alternative pathways and leads to the recruitment of circulating monocytes/macrophages [109].

### Complement system and oxidative stress in AMD

Recently, several research studies have identified various non-canonical functions of complement system factors, highlighting a mutual relationship between complement system and other pathomechanisms of AMD, such as oxidative or metabolic stress. Most studies have been performed on RPE cell lines, exposed to a variety of stress stimuli to mimic AMD adverse circumstances, and the levels of complement proteins and complement activation were analysed. Unfortunately, the different conditions and cell lines used in vitro has led to the generation of diverse results. For example, ARPE19 cells grown as a polarised monolayer, and subsequently exposed to H_2_O_2_, show an increase in gene expression and intracellular protein accumulation of complement proteins such as C3, C5, CR3, C5aR1, properdin (FP), Cathepsin B (CTSB) and Cathepsin L (CTSL): all independent of external complement supply [110]. Under the same conditions, FH was retained intracellularly with reduced cellular secretion even though no changes in *CFH* gene expression were reported [110]. In contrast, ARPE19 cells that are non-polarised and exposed to H_2_O_2_, show a reduction in gene expression of *CFH*, and intracellular and extracellular reduction of the FH protein, accompanied by extracellular accumulation of C3 [111, 112]. On the other hand, non-polarised hTERT-RPE1 cells show no gene regulation of *C3* or *CFH* in response to oxidative stress, no changes in C3/C3b in the supernatants and only a mild reduction of extracellular FH protein levels [113]. In addition, when ARPE19 cells are exposed to smoke extracts, as a source of oxidative stress, gene expression levels and extracellular levels of C3 were both increased, independent of the polarised monolayer status of the cells [114, 115]. Moreover, in response to smoke exposure, ARPE19 cells upregulate positive regulators of the complement system such as *CFB* and *C5* and reduce the protein levels of negative regulators, as CD55, CD59 and CD46 [114]. Similarly, exposure to products of lipid oxidation, such as Hydroxynonenal (HNE) and 4-Hydroxy-7-oxo-5-heptenoic acid (HOHA)-lactone or hypoxia leads to increase in C3 levels in both ARPE19 cells and induced pluripotent stem cells (iPSC)-derived RPE cells [116–118]. Moreover, HOHA-lactone leads to a general increase in ARPE19 in complement protein levels (C5, FB, CD55, CD59, CD46) [117]. Studies reported increased formation of C5b-9 complex formation on ARPE19 cells in response to different stressors [116, 118], indicating complement activation independent of external complement sources, while others show accumulation of MAC in response to smoke or HOHA-lactone in ARPE19 cells, only when supplemented with normal human serum (NHS) [114, 117]. Others reported undetectable levels of sC5b-9 in hTERT-RPE1 cells [113]. Recently, a cell-type specific screening of complement expression in retinal cells show the presence of most complement gene transcripts, but not all resulted in complete translation at the protein level, including C5, C8 and C9 [87].

As indicated above, the relationship between the complement system and cellular stress is mutual, i.e. not only are complement levels changed in response to stress, but also that a functional complement system is needed for a proper cellular response to stress. FH, either from local or systemic sources, plays a protective role against oxidative stress in RPE cells and this non-canonical function is altered by AMD-associated polymorphisms of *CFH* [113, 116]. Indeed, exogenously applied recombinant FH protein protects ARPE19 cells and iPSC-derived RPE cells from HNE-mediated damage, reducing the levels of cell death, complement activation, membrane and mitochondrial damage [116]. This observed protective effect was absent when repeated with FH carrying the AMD-risk variant 402H [116]. Moreover, FH has been found to be present in human plasma in two different redox forms, with the reduced form higher in early AMD patients and the oxidised form being higher in late AMD patients [119]. Interestingly, the two redox forms have different functions: the oxidised form mediates more efficiently FI-mediated cleavage of C3, while the reduced form protects ARPE19 cells from oxidative damage [119]. Furthermore, loss of endogenous FH in hTERT-RPE1 cells renders RPE cells more vulnerable to oxidative stress, leading to increase lipid peroxidation and reduced viability [113]. A chimeric transgenic mouse strain, carrying the *CFH* 402H, shows increase retinal oxidative stress, via malondialdehyde (MDA) and deposits accumulation, in response to ageing, light exposure and hydroquinone diet [120]. Beside FH, modulation of other complement components influences age or stress response of the retina. C3 depletion in mice ameliorates the function and structure of the aged retina, resulting in higher retinal thickness and light response, higher levels of antioxidants and lower levels of oxidative stress markers [121]. In a *Cfd*^−/−^ mouse model, exposure to constant light did not damage the retina as in *Cfd*^+/+^ counterpart, indicating that the alternative complement pathway may be involved in retinal damage mediated by constant light exposure [122].

### Complement system and lipids accumulation in AMD

The connection between complement and lipids goes beyond the involvement of complement system proteins in the protection from oxidised lipid products. Accumulation of lipids plays a fundamental role in AMD pathogenesis, as increased lipid deposition within Bruch’s membrane is known to occur in the early stages of AMD and indeed lipids are major components of drusen [47] (see Fig. 3). Dysregulation of the complement system or high-risk polymorphisms within complement genes has been associated with lipid accumulation, systemically or locally in the retina [123–125]. The 402H FH AMD-risk variant has been associated with lipoprotein accumulation (apolipoproteins B48 and A1 in particular) in the RPE/choroid of aged mice and who have been fed with a high-cholesterol diet [124]. In the same conditions, no differences in complement activation were recorded, implying a non-complement dependent function of the FH 402H variant in AMD pathogenesis [124]. iPSC-derived RPE cells carrying the *CFH* 402H polymorphism showed increased size of intracellular lipid globules compared to iPSC-derived RPE cells carrying *CFH* 402Y polymorphism [125].

Metabolomic studies performed on plasma/serum of AMD patients, showed a strong association between increased large and extra-large HDL levels and decreased VLDL and amino acid levels which were associated with increased complement activation, independent of AMD status [123]. Moreover, complement proteins including FH have been found in large HDL particles, while not present in small or medium HDL particles [126]. Whether the presence of FH in HDL particles is protective or not it remains elusive. On one hand it is hypothesised that an increased recruitment of FH in HDL particles would reduce the available circulating levels of FH, therefore increasing complement activation [123]. However, conversely the presence of FH in those particles could suppress an inflammatory response. In support of this second hypothesis, it has been shown that FH binds native LDL and with stronger affinity oxidised LDL (oxLDL) and this binding affinity is reduced in the FH high risk variant 402H [127]. FH binding to oxidised lipids is important in the modulation of inflammatory response in RPE cells and macrophages. Oxidised LDL leads to the upregulation of inflammatory cytokines C–C Motif Chemokine Receptor 2 (CCR2), IL8, TNF and VEGF and the binding of FH to those oxLDL mitigates the inflammation [127]. This function is abolished in the presence of the 402H variant or in the presence of the FHR proteins, which are antagonists of FH [127, 128] (see Fig. 3). Moreover, FH reduced the uptake of oxLDL by ARPE19 cells, possibly reducing their accumulation and this function was also abolished by the presence of FHRs [128].

### Complement system and energy metabolism in AMD

Disturbed RPE cell homeostasis, and in particular RPE cell metabolism, has been recently introduced as an important aspect of AMD pathology [129]. In fact, the energy metabolism of primary RPE cells isolated from AMD patients was found to be strongly impaired compared to RPE cells from healthy controls [130]. Recent studies highlighted an association between dysregulation of the complement system, mostly involving FH, and misbalance of energy metabolism, in particular in RPE cells and photoreceptors. Also, the absence of FH in the *Cfh*^*−/−*^ knock out mice influences retinal development [131]. In this model, the mitochondria of photoreceptor and RPE cells were found to be abnormally large, while mtDNA levels were reduced, indicating mitochondrial dysfunction, further confirmed by a decline in ATP production in *Cfh*^−/−^ mice [131]. Mitochondrial damage associated with complement system alterations have been shown in several models of RPE cells. RPE cells from AMD donors carrying the high risk 402H variant had more mtDNA damage compared with RPE from donors having the 402Y genetic variant [132]. iPSC-derived RPE cells carrying FH 402H polymorphisms present a lower number of mitochondria, but no differences in mitochondrial area, which indicates the presence of enlarged mitochondria [125]. FH loss in hTERT-RPE1 cells alters energy metabolism, including the reduction of mitochondrial respiration and glycolysis [113] leading to a phenotype similar to the one observed in primary RPE cells derived from AMD patients [130]. As for an oxidative stress response, the relationship between energy metabolism and complement system regulation appear to be mutual. In a cybrid model, consisting of mitochondria from AMD patients and ARPE19 deprived of mitochondria, changes in components of the complement pathway were observed [133]. Those changes involved an increase in gene transcription of complement activators (*CFP*, *CFB*, *CFHR4* and *CFHR1*) and a decrease in the transcription of complement inhibitors (*CFH*, *CD55*, *CD59*, *CFI* and *CD46*) [133].

Another vital aspect for RPE homeostasis is the autophagy–lysosomes machinery, which is responsible for the recycle of metabolites and degradation of damaged organelles (called mitophagy in the case of mitochondria) [134, 135]. Not surprisingly, dysfunction of the autophagy–lysosomes axis and mitophagy have been associated with AMD [136, 137]. While a connection between the complement system and autophagy–lysosomes pathway has been shown in kidney [138], pancreas [139] and T cells [140], it has only been shown recently in AMD models. iPSC-derived RPE cells carrying the FH 402H polymorphism show increased C5b9 deposition on the lysosomes, resulting in organelle swelling and reduced membrane integrity, indicator signs of malfunction [141] (see Fig. 3). Moreover, FH loss in hTERT-RPE1 cells resulted in increased gene expression of factors regulating mitophagy, PTEN Induced Kinase 1 (PINK1) and Parkin (PRKN) [113]. In a mouse model, depletion of C3 ameliorates autophagic flux in RPE cells, which was impaired with age progression [121].

## Complement-mediated therapeutic strategies for treating AMD

Given the strong genetic and biological evidence that over activation of the complement system is a major driver of AMD pathogenesis, it is perhaps not surprising that there is increasing interest in the complement-mediating therapeutic space, see Table 1 [142–145]. The current understanding suggests that AMD therapies should be directed towards regaining control of aberrant complement turnover locally within the macula, rather than focusing on systemic complement regulation. Indeed, early clinical trials with intravenously delivered Eculizumab, which targets C5 and is licensed for use in Paroxysmal nocturnal haemoglobinuria (PNH) and Atypical haemolytic uremic syndrome (aHUS) failed to show any effect in GA patients during a phase II clinical trial (NCT00935883) [146]. Several factors may have hampered successful Eculizumab treatment of GA, including the lack of stratification of patients based on their genetic risk, the trial intervening too late in the disease process or low drug dosage at the site of disease (i.e. the ECM of the choriocapillaris). Certainly, delivery of therapeutics directly into the eye holds a number of advantages and is now the preferred route of delivery with all current clinical trials in this space (Table 1).

However, delivery of complement-mediated therapeutics directly into the eye introduces a number of new hurdles to overcome. For example, regular intravitreal injections (such as those used for current anti-VEGF treatments for wet AMD) run the risk of introducing bacterial infections into the immune-privileged eye, where an active complement system plays a significant role in protecting the organ from such insults [147]. Therefore, regularly introducing into the eye an active biologic aimed at halting the complement system entirely (by perhaps eliminating the function of C3), using the technique primarily responsible for also introducing bacteria into the eye, would likely lead to an increased rate in inflammation and could result in endophthalmitis, a devastating diagnosis in ophthalmology [148]. Similarly, once inside the vitreous, any therapeutic would have to reach the appropriate anatomical site to have a desired effect, which might include the RPE, Bruch’s membrane or choriocapillaris and achieve an effective concentration over a sustained period of time.

One possible solution to the dosing question may be the delivery of therapeutics by gene therapy, i.e., targeting the RPE cell layer to produce the therapeutic, thus providing a constant locally delivered dose. The use of adeno-associated virus (AAV) vectors as a platform for gene therapy delivery of therapeutics has made a number of advances over the last few years [149] with Luxturna, an AAV-delivered gene therapy, getting FDA approval for the treatment of the rare inherited eye disorder, lebers congenital amaurosis (LCA) [150]. However, in the case of Luxturna the therapy is replacing the function of the *RPE65* gene within RPE cells, while in the case of AMD the therapeutic would likely be secreted and act elsewhere in the eye tissue. With this in mind, given that the primary site of complement over-activation is in the ECM underlying the RPE cells and Bruch’s membrane, it would be prudent to ensure that, presuming the therapeutic protein is basolaterally secreted, it is able to freely diffuse across Bruch’s membrane and does not become trapped at the RPE/Bruch’s membrane interface. Only the native soluble complement regulators FHL-1 and FD can diffuse through Bruch’s membrane given their small size and lack of glycosylation [37], whereas other complement regulators (e.g., FH or FI) cannot and may accumulate leading to damaging effects.

The point within the cascade at which complement needs to be targeted to achieve the most desired results also remains debated. The two most developed therapeutics (both currently in phase III trials), APL-2 and Zimura, target C3 and C5, respectively (see Table 1). Both prevent the formation of the C5a anaphylatoxin and the terminal complement pathway (i.e., MAC formation and cell lysis), but as APL-2 acts further up-stream on the C3 protein, it too prevents the formation of the C3a anaphylatoxin. Both APL-2 and Zimura reported a reduction GA lesion growth of 29 and 27.8% during their respective phase II trials, although both also saw an increased incidence of CNV in the treated eyes over sham [151, 152]. The increased incidence of CNV may be partly due to the pegylated (PEG) formulation of both therapies, as PEG has been used in the past to induce CNV in mouse models [153]. Nevertheless, APL-2 and Zimura remain the first complement-mediated therapeutics to show some form of efficacy in GA trials. Other therapeutic strategies currently under investigation include targeting regulators of the complement system, rather than the central C3 or C5 components (see Table 1). IONIS pharmaceutical’s targeting of *CFB* gene transcription in the liver will lead to a systemic reduction of this protein essential for forming the C3-convertase and feeding into the amplification loop of complement (Fig. 1). Again, it remains to be seen if systemic downregulation of complement activation will be efficacious for an eye disease, but the non-invasive subcutaneous delivery of the siRNA therapeutic has major advantages in terms of delivery methodology. Indeed, the delivery of siRNA to reduce liver expression of other complement genes, such as to reduce circulating levels of FHR proteins for example [154], may well be seen as suitably strategies in the future.

Genetic variants in the genes of FH and FI are being targeting through supplementation strategies (see Table 1). Gemini Therapeutics are currently undertaking a phase I clinical trial investigating the delivery of a FH biologic in an attempt to overcome reduced FH function in genetically susceptible GA patients (i.e., those carrying chromosome 1 genetic risk). Similarly, Gyroscope Therapeutics are targeting individuals who carry a rare genetic variant that leads to reduced circulating levels of FI [155, 156]. In this case, the FI therapeutic is being delivered by gene therapy to the RPE cells, such that it will be made locally. Neither FH nor FI cross Bruch’s membrane in their native forms [37], so it remains to be seen if enough of these therapies reach the underlying ECM of the choriocapillaris. Also, both strategies require the strict stratification of patients in order to target those who will be most sensitive to the respective treatments, something which has been postulated to have contributed to the failure of previous clinical trials, such as the anti-FD antibody Lampalizumab (Table 1). Another gene therapy-based approach is the over-expression of CD59 in RPE cells as being trialled by Hermera Biosciences. The subsequent increased level of membrane bound CD59 will reduce the amount of MAC being deposited on the RPE cells, causing cell lysis, and at sub lytic levels, inflammation [157].

Curiously, none of the strategies currently in clinical trials immediately address the down-stream consequences of complement activation, such as accumulation of circulating immune cells, which are present in large numbers in the back of an AMD eye [43]. At the point of therapeutic intervention, the ECM of the choriocapillaris and surrounding cells will already be coated in the opsonin C3b and iC3b allowing engagement of macrophage and/or microglia via their CR3 receptor [158], perpetuating phagocytosis, tissue damage and inflammation. One should also consider that at the time of therapeutic intervention, RPE cells have already undergone degeneration and, for example, may not be responsive to a specific gene therapy. Therefore, it is reasonable to take into consideration a combinational therapy targeting complement system and RPE cells at the same time. For instance, several antioxidants agents and potential mitochondrial protective substances have been suggested as cytoprotective options for RPE cells in AMD [159, 160].

## Summary

Aberrant complement activation, driven by a range of compounding genetic and environmental risk factors, clearly plays a significant role in the progression of AMD. It is also reasonable to suggest that targeting an overactive complement response in the back of the eye is a genuine strategy to slow down the progression of GA associated with dry AMD, especially given the recent advances made in clinical trials. However, it would be disingenuous to suggest that complement activation is the be-all and end-all when considering underlying mechanisms contributing to the diverse progression and physical manifestations of AMD.

So far, large international consortia on AMD such as the AMD Gene consortium (AMDGene: *N* = 50,000), the Three Continent Consortium (3CC; *N* = 35,000) and the EU Eye-RISK consortium (www.eyerisk.eu) have been very successful in the identification of genetic risk and environmental risk factors. However, due to lack of knowledge on the functional consequences of risk factors, the lack of appropriate animal models for AMD, and a lack of means to investigate functionality in humans, these consortia could not significantly extend investigations beyond risk identifiers.

Although the major genetic risks are defined, we currently do not know, why and how risks combine to advance progression in specific patients. Concerning risks associated to the alternative complement pathway, we still do not know how these risk alleles affect cellular physiology, whether they are independent drivers or part of a unified pathological scenario affecting the retina. The major risks, *CFH* mutations and *ARMS2/HTRA1* variant alleles present independently, yet we know little about their possible physiological interaction on a systems level or whether these two independent risk pathways converge on a higher order biological output or shared disease mechanism in the disease process. Different scenarios are possible: AMD could be considered at least in part as a complement disorder or as a Bruch’s Membrane disorder or as a deposit disease. It could also be seen as an RPE disease or common form of photoreceptor degeneration. Yet, we have reasons to believe, that risks interlink on the level of influencing physiological activity on a systems level, both locally and systemically [161]. For future research on AMD, combining different levels of molecular analysis (multi-omics) with multi-scale data integration and analysis supported by machine learning may open new ways of understanding the pathophysiology of AMD as a complex disease and deliver insights to explain the interplay of inherited and acquired risks for developing this devastating blinding disease.
